# Synthesis of Zwitterionic Copolymers via Copper-Mediated Aqueous Living Radical Grafting Polymerization on Starch

**DOI:** 10.3390/polym11020192

**Published:** 2019-01-22

**Authors:** Yifei Fan, Nicola Migliore, Patrizio Raffa, Ranjita K. Bose, Francesco Picchioni

**Affiliations:** Engineering and Technology Institute Groningen, University of Groningen, Nijenborgh 4, 9747AG Groningen, The Netherlands; y.fan@rug.nl (Y.F.); n.migliore@rug.nl (N.M.); p.raffa@rug.nl (P.R.); r.k.bose@rug.nl (R.K.B.)

**Keywords:** Cu^0^-mediated living radical polymerization, zwitterionic polymer, starch copolymer, anti-polyelectrolyte behavior

## Abstract

[2-(Methacryloyloxy)ethyl]dimethyl-(3-sulfopropyl)ammonium hydroxide (SBMA) is a well-studied sulfobetaine-methacrylate as its zwitterionic structure allows the synthesis of polymers with attractive properties like antifouling and anti-polyelectrolyte behavior. In the present work, we report the Cu^0^-mediated living radical polymerization (Cu^0^-mediated LRP) of SBMA in sodium nitrate aqueous solution instead of previously reported solvents like trifluoroethanol and sodium chloride aqueous/alcoholic solution. Based on this, starch-g-polySBMA (St-g-PSBMA) was also synthesized homogeneously by using a water-soluble waxy potato starch-based macroinitiator and CuBr/hexamethylated tris(2-aminoethyl)amine (Me_6_TREN) as the catalyst. The structure of the macroinitiator was characterized by ^1^H-NMR, ^13^C-NMR, gHSQC, and FT-IR, while samples of PSBMA and St-g-PSBMA were characterized by ^1^H-NMR and FT-IR. Monomer conversion was monitored by ^1^H-NMR, on the basis of which the reaction kinetics were determined. Both kinetic study and GPC results indicate reasonable controlled polymerization. Furthermore, a preliminary study of the thermal response behavior was also carried through rheological tests performed on aqueous solutions of the prepared materials. Results show that branched zwitterionic polymers are more thermal-sensitive than linear ones.

## 1. Introduction

Zwitterionic polymers, also known as polyzwitterions or polybetaines, are a special group of polymers containing equimolar amounts of both cationic and anionic sites in each repeat units. Accordingly, unlike conventional polyelectrolytes, which are charged when dissolved in water, zwitterionic polymers bear zero net charge under normal conditions. This special structure endows zwitterionic polymers with many properties uniquely different from normal water-soluble polymers. Among these, their anti-polyelectrolyte behavior is well known. It results in the solubility of zwitterionic polymers in saline water with the corresponding viscosity values being much higher than those in pure water because of the electrostatic screening effect [1,2,3]. This makes zwitterionic polymers attractive candidates for oil exploration such as drilling and enhanced oil recovery (EOR) [3,4,5]. Moreover, in recent years zwitterionic polymers are also recognized as potential materials for antifouling [6,7], drug carrier [8], organic electronic devices [9], and repeatable and reversible adhesion [10].

Generally, there are two pathways for the synthesis of zwitterionic polymers, one being the direct polymerization of zwitterionic monomers while the other requires post-polymerization modification [4,7,11]. Compared with the latter, direct polymerization of zwitterionic monomers has fewer side reactions and can easily yield electrically neutral polymers [4]. However, the selection of solvent is still challenging because of the poor solubility of polyzwitterions in most common solvents [4]. According to the literature, dimethyl sulfoxide (DMSO) [6,12], saline water [13,14], mixtures of water and alcohol [15,16] as well as trifluoroethanol (TFE) [9,17] have been utilized for the synthesis of zwitterionic polymers. Although saline water (mainly sodium halide salt solution) can be used for the synthesis of water-soluble polymers, the exchange of halide does affect the polymerization process [14,18]. This represents a serious limitation to the preparation of this class of polymers and makes it particularly difficult to find the right conditions to synthesize hydrophilic zwitterionic copolymers for applications like EOR and antifouling [3,4,5,6]. 

Typically, zwitterionic polymers are synthesized by free radical polymerization [5,19,20]. To investigate the influence of chain length and structure on the polymer properties, controlled polymerization like reversible addition-fragmentation chain transfer (RAFT) and transition metal-mediated reversible-deactivation radical polymerization (RDRP) have been reported in recent years [9,11,13,17,21,22]. Most of the controlled polymerizations were carried out with TFE, saline water or water/alcohol mixture as solvent. Compared with RAFT, which usually should be carried out above 50 °C, Cu^0^-mediated aqueous living radical polymerization (Cu^0^-mediated LRP) could be achieved at room temperature or even lower (0 °C for example) while maintaining a much higher polymerization rate [14,23]. However, reports on Cu^0^-mediated LRP of zwitterionic polymers are still rare compared with RAFT and normal ATRP [12,13,17,24,25,26,27]. So far as we know, only [2-(methacryloyloxy)ethyl]dimethyl-(3-sulfopropyl)ammonium hydroxide (SBMA) was polymerized in NaCl aqueous/ethanol solution with chloride as the functional group by Cu^0^-mediated LRP [14]. 

In order to expand the possibilities of preparing zwitterionic copolymers, also focusing on the “green” aspects, in the present work SBMA was polymerized by Cu^0^-mediated LRP in sodium nitrate aqueous solution with bromide as the functional group. Compared with the commonly used sodium halide salts, sodium nitrate has less influence on the polymerization [14,18]. Based on this, in order to compare the properties of structurally different polyzwitterions, bio-based starch-g-polySBMA (St-g-PSBMA) was also homogeneously synthesized from waxy starch-based macroinitiator. The polymerization kinetic was monitored by ^1^H-NMR and the product was characterized by Fourier transform infrared (FT-IR). The anti-polyelectrolyte behavior and thermal response property in water solution of the synthesized polymers were also investigated with a rheometer. To the best of our knowledge the graft polymerization on starch as well as the chosen reaction conditions (in water) constitute relevant novelty of the present work. Moreover, both factors help in framing the present research in a general context of green materials prepared through green processes. 

## 2. Materials and Methods 

### 2.1. Materials

Waxy potato starch (>95% amylopectin) was kindly donated by Avebe (Veendam, The Netherlands) and dried under vacuum at 60 °C for 48 h before use. Lithium chloride was purchased from Sigma–Aldrich (Steinheim, Germany) and dried under vacuum at 80 °C for 24 h before use. Anhydrous *N*,*N*-dimethylacetamide (DMAc) was purchased from Sigma–Aldrich in Sure/Seal™ (Steinheim, Germany). 2-bromopropionic acid (BpA), 2-bromopropionyl bromide (BpB), formaldehyde solution (37%), and formic acid (>95%) were purchased from Sigma–Aldrich (Steinheim, Germany) and used as received. Tris(2-aminoethyl)amine (TREN) was purchased from Tokyo Chemical Industry Co., LTD (TCI, Tokyo, Japan) and used as received. Tris[2-(dimethylamino)ethyl]amine (Me_6_TREN) was synthesized following the procedures reported [28] and [2-(Methacryloyloxy)ethyl]dimethyl-(3-sulfopropyl)ammonium hydroxide (SBMA) (97%) was purchased from Merck (Darmstadt, Germany) and used as received. Copper(I) bromide (from Sigma-Aldrich) was stirred in sulfuric acid (from Sigma–Aldrich) solution for 2 h, then filtered, washed with ethanol and acetone respectively three times and dried under vacuum at room temperature for 24 h, followed by storage under N_2_ atmosphere.

### 2.2. Characterization

NMR spectra were recorded on Varian Mercury Plus 400 MHz spectrometer (Varian, Inc., Palo Alto, CA, USA) using deuterated solvents purchased from Sigma–Aldrich. Fourier transform infrared spectra were recorded with attenuated total reflection (ATR) accessories on an IRTracer-100 SHIMADZU Fourier transform infrared spectrophotometer (Shimadzu Corp., Kyoto, Japan) and data were processed with LabSolutions IR software (Version 2.11, Shimadzu, Kyoto, Japan, 2014). Aqueous gel permeation chromatography (GPC) was conducted on a Jasco PU-1580 HPLC pump system (JASCO, Easton, MD, USA) equipped with a Jasco RI-2031 differential refractive index (DRI) detector and two 300 mm × 7.8 mm Ultrahydrogel Linear column sets from Waters (Milford, MA, USA). The mobile phase used was 1 M NaNO_3_ and the pH was adjusted to 7.4 with phosphate-buffered saline (PBS) buffer tablet purchased from Sigma–Aldrich (Steinheim, Germany). Column oven and detector temperatures were regulated to 25 °C, with a flow rate of 0.8 mL/min. Monodispersed polyethylene glycol (PEG) standards from Sigma–Aldrich were used for calibration. Samples were filtered through a membrane with a 0.22 μm pore size before injection. Experimental molar mass and polydispersity index (PDI) values of synthesized polymers were determined by conventional calibration using JASCO Borwin GPC software (JASCO, Easton, MD, USA). Rheology properties were measured with a HAAKE Mars III (Thermo Scientific, Waltham, MA, USA) rheometer equipped with a cone-plate geometry (diameter 60 mm, angle 2°). Solution viscosity was measured as a function of shear rate (0.1 to 1750 s^−1^, T = 20 °C), salt concentration (2500–300,000 ppm of NaCl, T = 20 °C, shear rate 9.6 s^−1^) and temperature (10 to 90 °C, shear rate 9.6 s^−1^), respectively, with a polymer concentration of 5 wt %.

### 2.3. Synthesis of Starch-Based Macroinitiator (StBr)

Waxy potato starch (2.59 g, 16 mmol) and lithium chloride (1.02 g, 24 mmol) were added to a 250-mL three-necked flask (dried overnight at 100 °C before use) connected with a mechanical stirrer. The system was vacuumed under heat and backfilled with N_2_ three times to remove residual water. Anhydrous dimethylacetamide (DMAc) (100 mL) was transferred to the flask and the mixture was stirred at 130 °C for about 1 h under N_2_ atmosphere. A transparent solution formed when the mixture cooled down to room temperature naturally. The solution was cooled down with an ice bath and then 0.42 mL (4 mmol) BpB was added dropwise within 30 min under the protection of N_2_. The mixture was then warmed up naturally to room temperature and stirred for 3 h. The final products were precipitated out with tenfold acetone and then filtered, washed, and dried under vacuum at 45 °C for 24 h. The resulting white powder was then purified by Soxhlet extraction with acetone as solvent for 24 h (final yield 87%).

### 2.4. Aqueous Cu^0^-Mediated LRP of SBMA

The polymerization of SBMA followed the procedures reported for the synthesis of polyacrylamide [29].

*Typical Polymerization Protocol.* H_2_O (2.5 mL) and Me_6_TREN (11.4 μL, 0.04 mmol) were charged to a 25-mL three-neck round-bottom flask with a magnetic stirrer bar and rubber septum. The solution was vacuumed and backfilled with N_2_ three times to remove O_2_. Copper (I) bromide (7.0 mg, 0.04 mmol) was added with rapid stirring. The solution was cooled down with an ice bath. Simultaneously, another 25-mL three-neck round-bottom flask was charged with BpA (15.3 mg, 0.1 mmol equiv. Br), SBMA (1.39 g, 5 mmol), sodium nitrate (0.85 g, 10 mmol), and 2.5 mL of Milli-Q water. The flask was degassed with three cycles of vacuum and backfilled in ice bath. This monomer/macroinitiator solution was then transferred to the flask containing catalyst solution via degassed syringe. The mixture was allowed to react for 25 min. The resulting solution was dialyzed against Milli-Q water and then freeze-dried for 24 h. Samples were named in the way like PSBMA38, the number 38 stands for the degree of polymerization (DP) of synthesized polymer.

Copolymers of St-g-PSBMA were prepared according to the same procedure with StBr as the initiator.

## 3. Results and Discussion

### 3.1. Synthesis of Waxy Potato-Based Macroinitiator StBr

The synthesis and characterization of StBr can be seen in the Supplementary Materials (Figures S1 and S2).

### 3.2. Aqueous Cu^0^-Mediated LRP of SBMA

**Figure 1:**
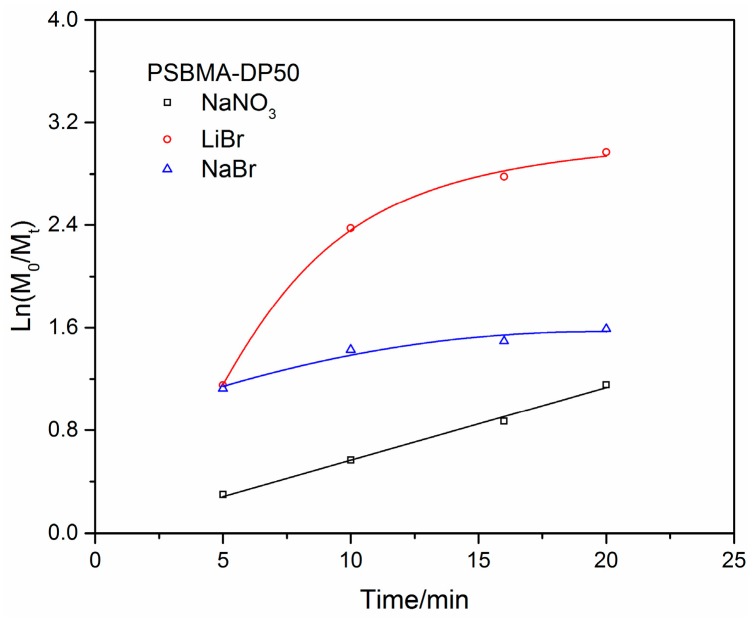
Kinetic plot of Cu^0^-mediated LRP of SBMA with target DP of 50 in different salt solution (the solid line is a guide to eye).

Although there is still a debate about the actual mechanism active in the process, which could be either a single-electron transfer living radical polymerization (SET-LRP) mediated by Cu^0^ or a supplemental activation reducing agent ATRP (SARA-ATRP) mediated by Cu^I^, the employed synthetic method (vide supra) has been proven to be efficient and successful in the polymerization of monomers like acrylamide in water [23,29,30]. According to a previous report [14], the addition of a halide salt (e.g., NaCl) is beneficial for the control of polymerization when chloride is used as the functional group for Cu^0^-mediated LRP of SBMA in the aqueous/ethanol mixture. In this paper, the influence of three different salts on the controllability of Cu^0^-mediated LRP with bromide as the functional group was preliminarily studied. Cu^0^-mediated LRP of SBMA was carried out with BpA as initiator and CuBr/Me_6_TREN as the catalytic system (see entry 2, Table 1, conditions are the same except the change of salt). The kinetic results (Figure 1) clearly indicate the loss of control on the polymerization when lithium bromide and sodium bromide is used (the concentration of salt is 1 M). However, a controllable polymerization route is indicated when sodium nitrate is used. The reasons for this are still not clear, but could be related to the higher reactivity of bromide compared with that of chloride [31]. Nevertheless, sodium nitrate was chosen for the following study because of its neutral role in the polymerization.

**Figure 2:**
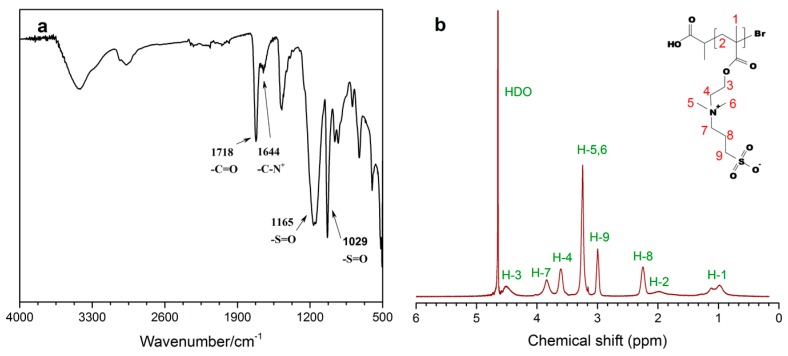
FT-IR (**a**) and ^1^H-NMR spectra (**b**, in D_2_O) of PSBMA.

A series of PSBMA with the DP ranging from 14 to 360 were synthesized (Table 1). The polymer was characterized by FT-IR (Figure 2a) and ^1^H-NMR (Figure 2b). The absorption peak at 1718 cm^−1^ in FT-IR spectrum was assigned to the stretch of C=O bond in the ester group [21]. The shoulder peak at 1644 cm^−1^ is the absorption from the stretch vibration of C–N^+^ bond [21]. The symmetrical and asymmetrical stretch of S=O bond in the sulfonate group could also be seen at 1029 and 1165 cm^−1^, respectively [21]. The ^1^H-NMR spectrum is shown in Figure 2b, in which the peaks in the range of 0.75–1.30 ppm were attributed to the methyl protons on the backbone of PSBMA. The peaks at 3.25, 3.61, and 3.84 ppm originate from the methyl and methylene protons in the quaternary amine group, respectively. Signals from the methylene protons connected to the ester and sulfonate groups could also be seen at 3.00 and 4.52 ppm. Moreover, signals from the proton of the double bond (between 5.5–6.2 ppm) in monomer were not found.

Due to the strong intra- and intermolecular electrostatic forces, only PSBMA polymer with low DP (thus good solubility in GPC eluent) was characterized with GPC (Table 1, entry 1 to 5). Nevertheless, for all the polymerization, the monomer conversion was monitored with ^1^H-NMR and the kinetic plot was thus obtained according to Equation (1) [32].

(1) ln(M0/Mt)=kp(Ri/kt)12·t

where *M*_0_ and *M*_t_ are the monomer concentration at the beginning of polymerization and at time *t*, respectively, $k_{p}$ indicates the kinetic propagation constant, $R_{i}$ the initiation rate, and $k_{t}$ the termination rate constant.

**Figure 3:**
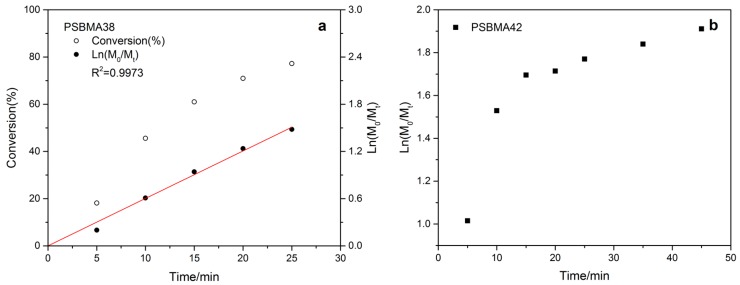
Kinetic plot of Cu^0^-mediated LRP of SBMA for target DP 50—the influence of CuBr to ligand ratio on kinetic and controllability.

As shown in Table 1, the initial homogeneous Cu^0^-mediated LRP of SBMA was carried out with a ratio of (M):(Initiator):(CuBr):(Me_6_TREN) of 25:1:0.4:0.4 (entry 1). A monomer conversion of 56.3% was achieved within 25 min with a linear kinetic curve (Figure S3) and a PDI of 1.42 which indicate a relatively controlled polymerization. When the targeted DP was increased to 50 (entry 3) with the same catalyst to initiator ratio, a monomer conversion of 77.2% could be achieved within 25 min and the kinetic study (Figure 3a) also shows reasonable control over the polymerization. This is in line with the GPC result as shown in Table 1. Doubling the ratio of CuBr resulted in a faster polymerization rate at the beginning, then a significant drop in the reaction rate was observed after 15 min (entry 4, Figure 3b). This was attributed to the accumulation of CuBr_2_ in the reaction system. This could be attributed to radical termination and a resulting buildup in the concentration of deactivator CuBr_2_ [32]. Termination itself can also be a cause of decreased rate. For the target DP of 100 and 200 (entry 5 and 6), a slight excess of CuBr to ligand was used to maintain sufficient deactivation as reported [23,29]. In this way, a conversion over 70% was achieved and the control over the polymerization was still maintained according to the kinetic study (Figures S4 and S5). However, when the same catalyst-to-initiator ratio was applied for the target DP 400, no apparent reaction was observed (entry 7). The monomer concentration was then increased from 1.0 to 1.5 M and 65.9% of the monomer was polymerized with reasonable control as indicated by the linear kinetic curve (entry 8, Figure S6). Considering the high viscosity during the reaction, the polymerization temperature was increased from 0 to 25 °C for the preparation of polymer with higher molecular weight (entry 9 and 10). To maintain a balance between the control over the polymerization and monomer conversion, the monomer concentration was decreased while the reaction time was prolonged. For entry 9, although the monomer conversion is relatively lower compared with entry 10 (Figures S7 and S8), a faster polymerization rate was observed due to the lower deactivator concentration as indicated by the monomer concentration in Table 1. Nevertheless, in both cases controlled polymerization was observed according to the kinetic monitoring.

**Figure 4:**
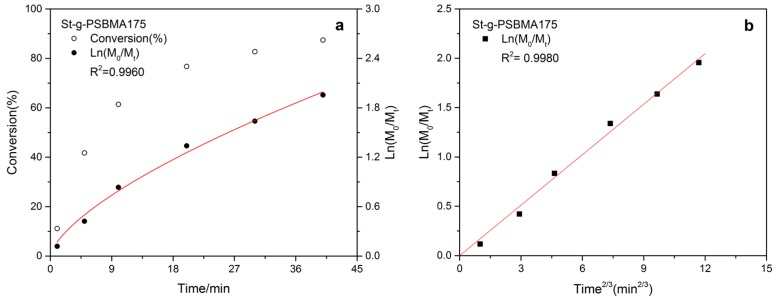
Kinetic plot for the synthesis of St-g-PSBMA for target DP 200.

As shown in Table 2, copolymers of PSBMA with starch (St-g-PSBMA) were also synthesized with Cu^0^-mediated LRP. The FT-IR and ^1^H-NMR spectra of the copolymer could be seen in Figures S9 and S10, respectively. It was noticed that the initial monomer concentration of 0.67 M was too low for the successful synthesis of copolymer when the ratio of CuBr to Me_6_TREN was set to 1.2:0.6 (entry 1, Table 2). Interestingly, when the monomer concentration was increased to 0.75 M (entry 2, Table 2), a power-law dependence plot of the conversion index (ln[M_0_/M_t_]) on time was obtained (Figure 4a). The kinetics of living radical polymerization, according to earlier publications [32,33], can be divided into stationary-state and power-law kinetics. Equation (1) represents the stationary-state kinetic which is applicable to systems with a relatively large initiation rate, while the power-law kinetic normally could be observed in systems with zero or a very low initiation rate [32]. In the present research, the low initiation rate may be attributed to the high initial deactivator concentration due to the disproportionation of CuBr (see Experiment part Section 2.4 and the initiator-to-CuBr ratio in Table 2). The power-law kinetic could be represented by Equation (2) [32].

(2) ln(M0/Mt)=32kp(KAT·I03·kt)13·t23

where *M*_0_ and *M*_t_ are the monomer concentration at the beginning of polymerization and at time *t*, respectively, $K_{AT}$ is the equilibrium constant stands for the ratio of the activation rate constant to the deactivation rate constant.

The kinetic for Cu^0^-mediated LRP of SBMA onto starch (entry 2, Table 2) was then modeled with equation 2 (Figure 4b). Clearly, a linear correlation between ln[M_0_/M_t_] and *t*^2/3^ was obtained. The same polymerization was also carried out with a monomer concentration of 0.85 M (entry 3, Table 2). Although a higher monomer conversion was achieved within a shorter time compared with entry 2 (Table 2), the deviation from linear on the kinetic plot indicated an uncontrolled polymerization (Figure S11). One possible reason for the loss of control could be the higher polymerization rate resulted from the higher monomer and catalyst concentration. Considering the lower concentration of initiator (as in the case of entry 6, 7, and 8, Table 1), when the target DP of PSBMA was increased to 400 the monomer concentration was set to 0.83 M instead of 0.75 M. A reasonable control over the grafting process with a high monomer conversion was observed according to the linear kinetic plot on a timescale of *t*^2/3^ (Figure S12).

**Figure 5:**
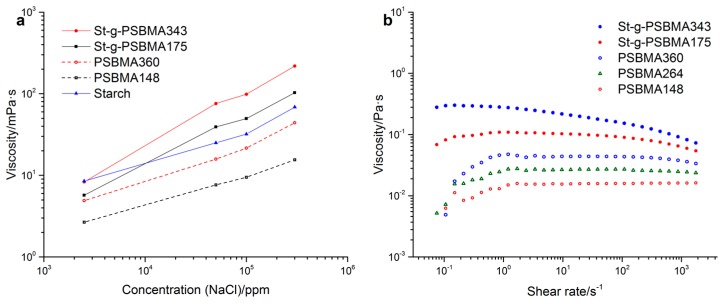
Solution viscosity vs. salt concentration ((**a**) shear rate 9.6 s^−1^) and solution viscosity vs. shear rate (**b**).

A preliminary study on the rheological properties of PSBMA and St-g-PSBMA was carried out at the same polymer concentration (5.0 wt %). The anti-polyelectrolyte property of synthesized polymers was studied in sodium chloride solution the concentration of which ranges from 2500 to 300,000 ppm. As shown in Figure 5a, the viscosity of all the samples increased with increasing salt concentration. The increase in the viscosity of starch solution was attributed to the “structure” change of water due to the addition of NaCl (e.g., the reducing of free water) [34,35]. For polyzwitterionic polymers, the addition of NaCl breaks the intramolecular electrostatic interaction, and thus a more extended polymer chain (larger hydrodynamic volume) results in the increase of solution viscosity [1,2]. Compared with PSBMA homopolymer, higher viscosity was observed on St-g-PSBMA copolymer with similar PSBMA chain length, especially at higher salt concentration. Moreover, higher increment ratio in the viscosity of St-g-PSBMA compared with corresponding homopolymer and starch indicated a faster increase in the hydrodynamic volume of copolymer with changing salinity, especially in the lower salinity range. These indicated a successful grafting of PSBMA on starch.

The viscosity of the polymer versus shear rate was measured in 300,000 ppm NaCl solution to ensure the complete dissolution of the polymer and the result is displayed in Figure 5b. As can be observed, the viscosity of homopolymer increased gradually with the increase of DP, which to some extent also indicates a controllable polymerization method. Higher viscosity (with a Newtonian plateau at low shear rates and shear-thinning behavior at high shear rates) was observed on St-g-PSBMA copolymer because of higher hydrodynamic volume compared with the corresponding PSBMA homopolymer [36,37].

**Figure 6:**
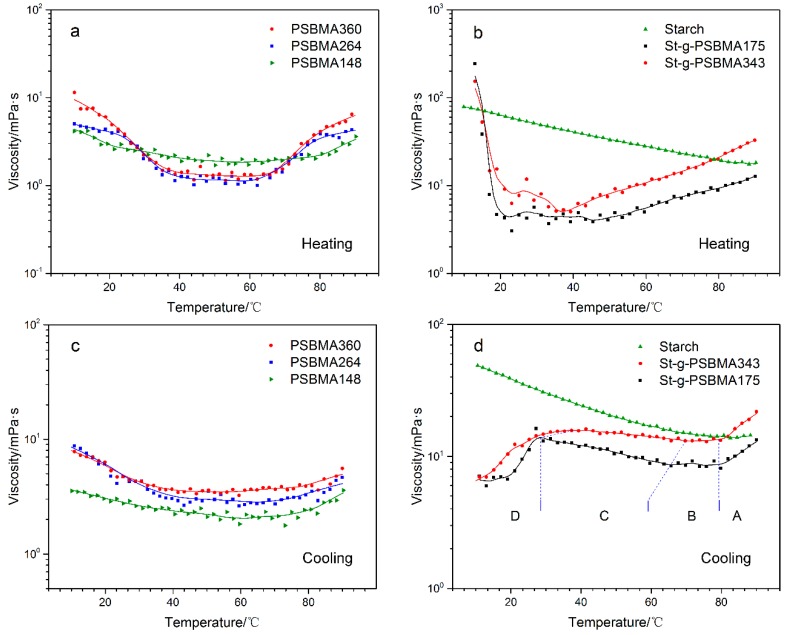
Solution viscosity vs. temperature of (**a**) PSBMA homopolymer (Heating), (**b**) St-g-PSBMA (Heating), (**c**) PSBMA homopolymer (Cooling), and (**d**) St-g-PSBMA (Cooling) (the solid line is to guide the eye).

Thermo-responsive property of the zwitterionic polymers is also of interest for potential applications like EOR and repeatable-reversible adhesion [3,10]. The thermo-responsive property of synthesized polymer solution was studied at the polymer concentration of 5.0 wt %. Figure 6a,b are the heating curves of viscosity versus temperature in the range of 10 to 90 °C while Figure 6c,d are the corresponding cooling curves. Clearly in Figure 6a, the viscosity of PSBMA homopolymer, with the same trend as that of starch (Figure 6b), dropped with increasing temperature. However, when the temperature was higher than 40 °C, a plateau was observed and then the viscosity increased as the temperature went up. This trend is more obvious for PSBMA polymer with higher DP. According to Figure 6b, the starting point (around 40 °C) of viscosity increase for St-g-PSBMA is much lower than that (around 60 °C) of PSBMA homopolymer. This implies that the copolymer is more thermo-sensitive than the homopolymer. In the cooling curve of PSBMA homopolymer (Figure 6c), contrary to that of starch solution (Figure 6d), the viscosity first dropped due to the collapse of molecule chains as the temperature decreased. When the temperature dropped below 50 °C, an increase in the viscosity was observed. Compared with the homopolymer, the cooling curve of St-g-PSBMA solution could be divided into four section as shown in Figure 6d. Given the highly branched structure of the copolymer (see Graphical Abstract), the viscosity decrease in Section A can be attributed to the collapse of molecule hydrodynamic volume due to the stronger intra/inter-chain electrostatic attraction at lower temperature. Upon further cooling, a dynamic equilibrium between the shrinking of molecular hydrodynamic volume and the increasing of intermolecular interaction achieved, thus a plateau (Section B) was seen in the cooling curve. After this stage, higher intermolecular interaction resulted in increased viscosity as observed in section C. When the temperature dropped below a critical point the polymer precipitated out of solution because of the strong intra- and inter-molecular interaction, as the result of which a drop in the viscosity was noticed in Section D. The critical temperature, according to the comparison of two different copolymers, increases as the PSBMA chain length grows longer. It should be noted that reports on the rheology behavior of highly branched PSBMA are still rare. In the present research, the proposed mechanism on the unusual viscosity-temperature profile is based on the upper critical solution temperature (UCST) behavior of linear PSBMA copolymer as reported [38,39]. More detailed study are needed to thoroughly understand this phenomenon.

## 4. Conclusions

In the present study, Cu^0^-mediated LRP of SBMA was carried out in sodium nitrate aqueous solution instead of commonly used TFE and sodium chloride aqueous/alcoholic solution. In contrast with sodium halides, sodium nitrate has less influence on polymerization. Linear kinetic plots obtained from ^1^H-NMR and GPC results revealed an acceptably controlled polymerization method. Tunability of Cu^0^-meditated LRP on the synthesis of polymers was also demonstrated by the flow curve. Starch-g-PSBMA was, for the first time, as far as we know, synthesized by aqueous Cu^0^-mediated LRP with waxy potato starch-based macroinitiator as well. The difference in the anti-polyelectrolyte behavior between the homopolymer and corresponding copolymer, according to the rheology test result, proved the successful synthesis of St-g-PSBMA. The sensitivity of the linear and branched polymer to the change of temperature could all be tuned by varying the chain length of polyzwitterions. The copolymer, compared with homopolymer as could be seen in the thermal rheology analysis, is more thermally sensitive due to the branched structure. This implies that branched zwitterionic polymers should be more suitable for applications like thermally controlled drug delivery and applications requiring controlled rheological behavior such as enhanced oil recovery.

## Figures and Tables

**Table 1:** Experimental data of Cu^0^-mediated LRP of SBMA.

Entry	[M]:[I]:[CuBr]:[L]	Time/min	Temperature/°C	Concentration *^a^*/mol⋅L^−1^	Conversion *^b^*/%	DP *^b^*	PDI *^c^*
1	25:1:0.4:0.4	25	0	1.0	56.3	14	1.38
2	50:1:0.4:0.4	20	0	1.0	71.6	36	1.50
3	50:1:0.4:0.4	25	0	1.0	77.2	38	1.33
4	50:1:0.8:0.4	45	0	1.0	85.2	42	1.73
5	100:1:0.8:0.6	25	0	1.0	75.9	76	1.40
6	200:1:0.8:0.6	25	0	1.0	74.3	148	- ***^e^***
7	400:1:0.8:0.6	25	0	1.0	- ***^d^***	- ***^d^***	- ***^d^***
8	400:1:1.0:0.6	25	0	1.5	65.9	264	-***^e^***
9	600:1:1.0:0.6	25	25	0.75	55.9	336	-***^e^***
10	600:1:1.0:0.6	60	25	0.85	60.0	360	-***^e^***

*^a^* Monomer concentration in the feeding solution; *^b^* Monomer conversion and degree of polymerization (DP) determined according to ^1^H-NMR; *^c^* Polydispersity index (PDI) obtained from GPC; *^d^* No reaction; ^*e*^ No PDI data due to the insufficient solubility of sample in GPC eluent.

**Table 2:** Experimental data of Cu^0^-mediated LRP of SBMA copolymer ***^a^***.

Entry	[M]:[I]:[CuBr]:[L]	Time/min	Temperature/°C	Concentration *^b^*/mol⋅L^−1^	Conversion *^c^*/%	DP *^c^*
1	200:1:1.2:0.6	25	25	0.67	- ***^d^***	- ***^d^***
2	200:1:1.2:0.6	40	25	0.75	87.4	175
3	200:1:1.2:0.6	25	25	0.85	90.2	180
4	400:1:1.2:0.6	40	25	0.83	85.9	343

*^a^* No polydispersity index (PDI) obtained from GPC due to insufficient solubility of sample in GPC eluent; *^b^* Monomer concentration; *^c^* Monomer conversion and degree of polymerization (DP) determined according to ^1^H-NMR; *^d^* No reaction.

## Supplementary Materials

The following are available online at http://www.mdpi.com/2073-4360/11/2/192/s1, Scheme S1: Synthesis of waxy potato starch-based ATRP macroinitiator and St-g-PAM, Figure S1: FT-IR spectra of St-Br with different DS, Figure S2: ^1^H-NMR (a), ^13^C-NMR (b) and gHSQC (c) spectra of St-Br (DS = 0.15) in D_2_O, Figure S3: Kinetic plot of Cu^0^-mediated LRP of SBMA with target DP of 25, Figure S4: Kinetic plot of Cu^0^-mediated LRP of SBMA with target DP of 100, Figure S5: Kinetic plot of Cu^0^-mediated LRP of SBMA with target DP of 200, Figure S6: Kinetic plot of Cu^0^-mediated LRP of SBMA with target DP of 400, Figure S7: Kinetic plot of Cu^0^-mediated LRP of SBMA with target DP of 600, Figure S8: Kinetic plot of Cu^0^-mediated LRP of SBMA with target DP of 600, Figure S9: FT-IR spectrum of Starch-g-PSBMA, Figure S10: ^1^H-NMR spectrum of Starch-g-PSBMA, Figure S11: Kinetic plot for the synthesis of St-g-PSBMA with target DP of 200, Figure S12: Kinetic plot for the synthesis of St-g-PSBMA with target DP of 400.
