# Supplementary material for: Synthesis of Zwitterionic Copolymers via Copper-Mediated Aqueous Living Radical Grafting Polymerization on Starch
Source: Polymers (Basel). 2019 Jan 22;11(2):192. doi: 10.3390/polym11020192 (PMC6418991; doi:10.3390/polym11020192)
Supplement: Supplementary file 1 [file polymers-11-00192-s001.pdf]

polymers

Supplementary Material: Synthesis of Zwitterionic (Co)Polymers via Copper-mediated Aqueous Living Radical Grafting Polymerization on Starch

Yifei Fan, Nicola Migliore, Patrizio Raffa, Ranjita K. Bose and Francesco Picchioni

1. The Synthesis of Starch-Based Macroinitiator

The starch based macroinitiator StBr was prepared homogeneously via one step esterification reaction with 2-bromopropionyl bromide (BpB) in DMAc/LiCl as depicted in Scheme S1. Both FT-IR (Figure S1) and NMR (Figure S2a ^{1}H-NMR, S2b ^{13}C-NMR, S2c gHSQC) were employed to demonstrate the successful preparation of the macroinitiator. The absorption peak at 1743 cm^{-1} in FTIR spectrum was assigned to the stretch of the C=O group from the initiator while the peak at 1281 cm^{-1} was attributed to the C–O bond in the ester group. The successful synthesis of StBr was further proved by NMR spectra. In Figure S2a, the peak at 5.4 ppm should be assigned to the proton attached to the anomeric carbon and peaks in the range of 3.3 - 4.2 ppm should be attributed to the rest protons of the anhydroglucose unit (AGU). Peaks around 1.7 ppm and 4.7 ppm belong to methyl protons and methine proton of the 2-bromopropionyl group, respectively. The degree of substitution(DS) of StBr could be quantified with the peak at 5.4 ppm and 4.7 ppm. In the ^{13}C-NMR spectrum (Figure S2b), the peak at 100 ppm belongs to the anomeric carbon and the peaks range from 60 ppm to 80 ppm should be assigned to the rest AGU carbons. The resonance of methyl and methine carbon locate at 22 ppm and 54 ppm respectively. These assignments are verified by the gHSQC spectrum (Figure S2c).

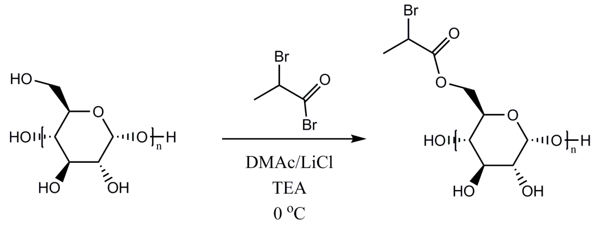

Scheme S1. Synthesis of waxy potato starch-based ATRP macroinitiator.

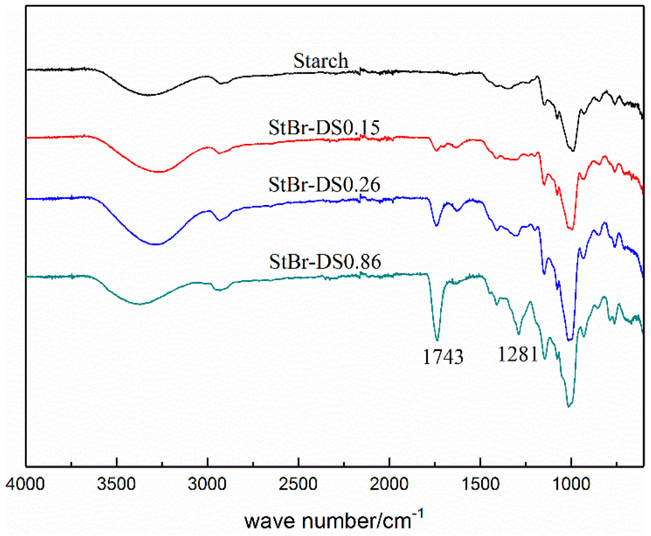

Figure S1. FT-IR spectra of StBr with different DS.

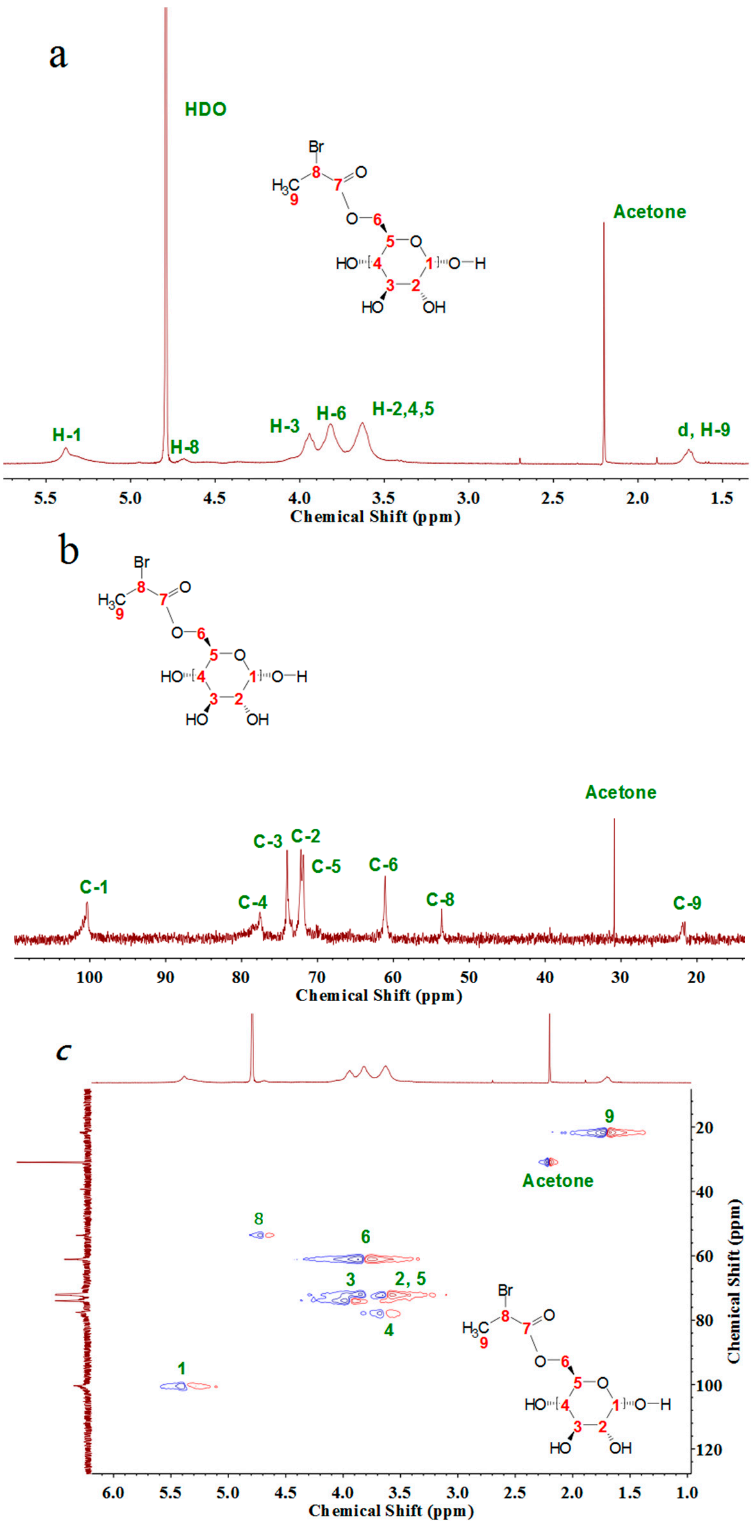

Figure S2. ^{1}H-NMR (**a**), ^{13}C-NMR (**b**) and gHSQC (**c**) spectra of St-Br (DS = 0.15) in D_2O.

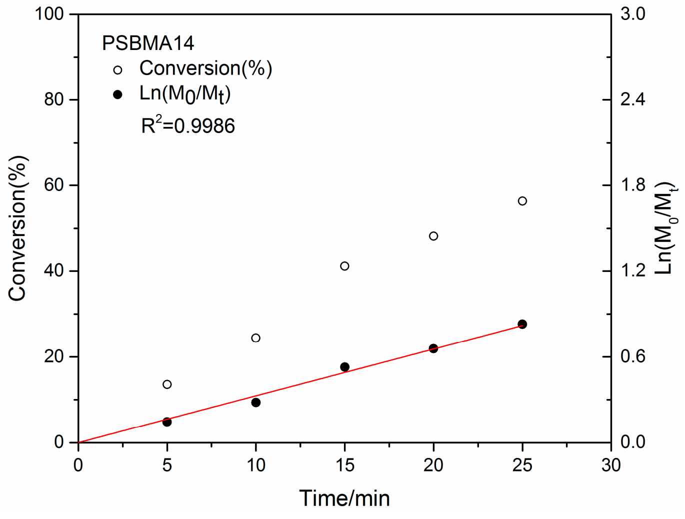

Figure S3. Kinetic plot of Cu^0-mediated LRP of SBMA with target DP of 25

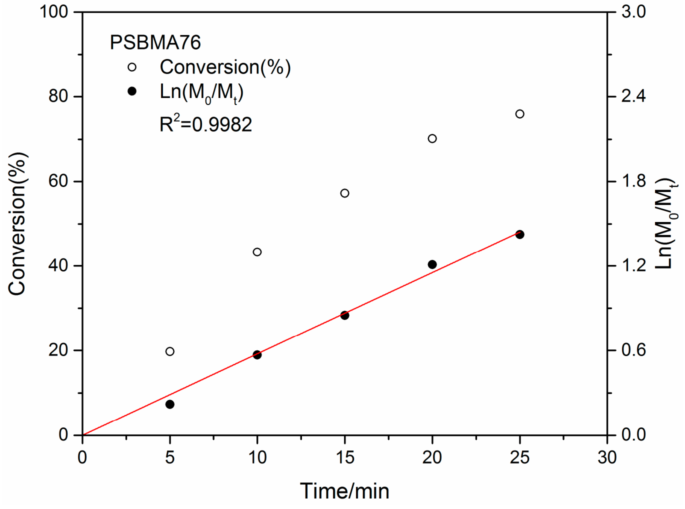

Figure S4. Kinetic plot of Cu^0-mediated LRP of SBMA with target DP of 100

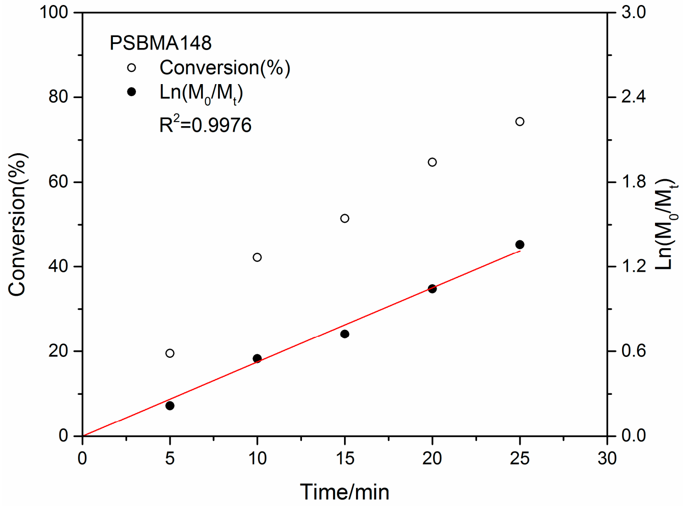

Figure S5. Kinetic plot of Cu^0-mediated LRP of SBMA with target DP of 200

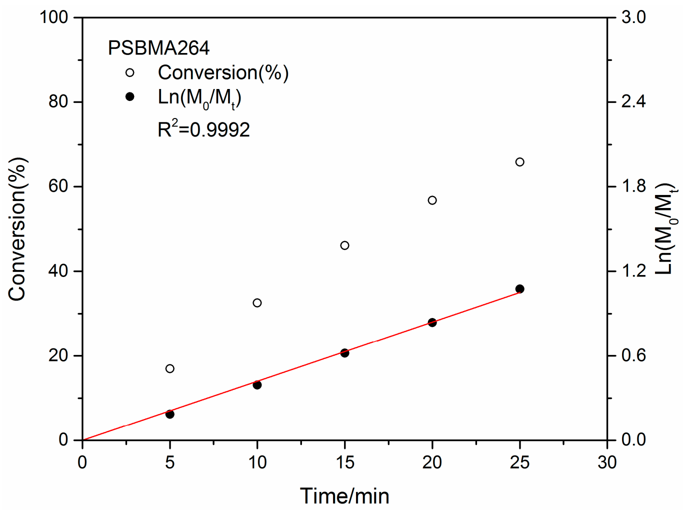

Figure S6. Kinetic plot of Cu⁰-mediated LRP of SBMA with target DP of 400

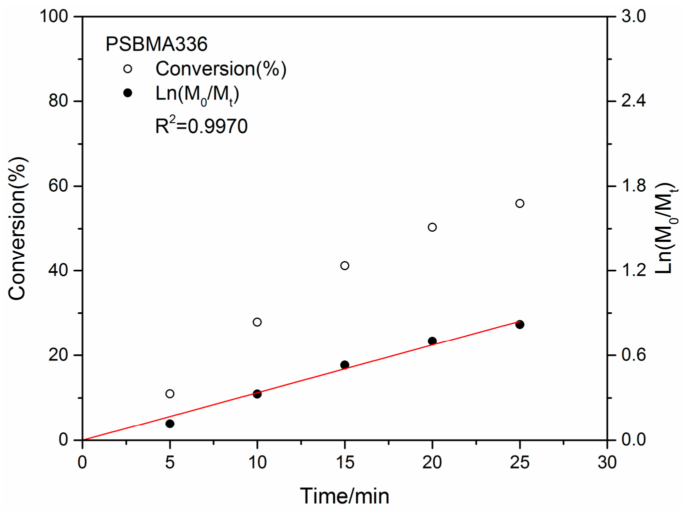

Figure S7. Kinetic plot of Cu⁰-mediated LRP of SBMA with target DP of 600 (entry 9)

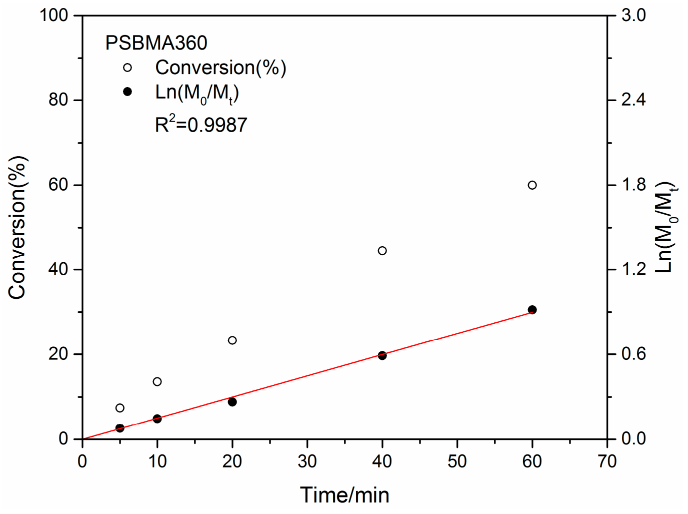

Figure. S8 Kinetic plot of Cu⁰-mediated LRP of SBMA with target DP of 600 (entry 10)

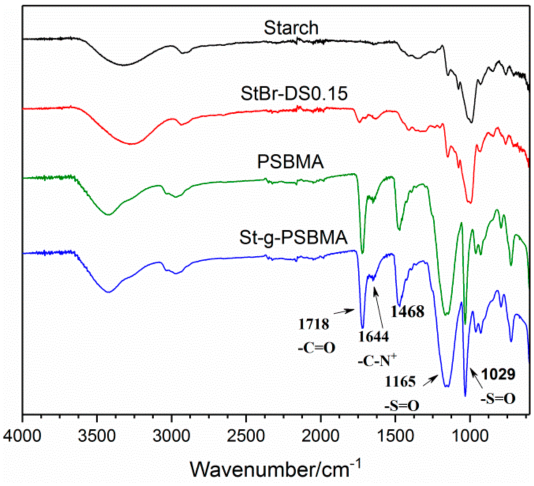

Figure S9. FT-IR spectrum of Starch-g-PSBMA

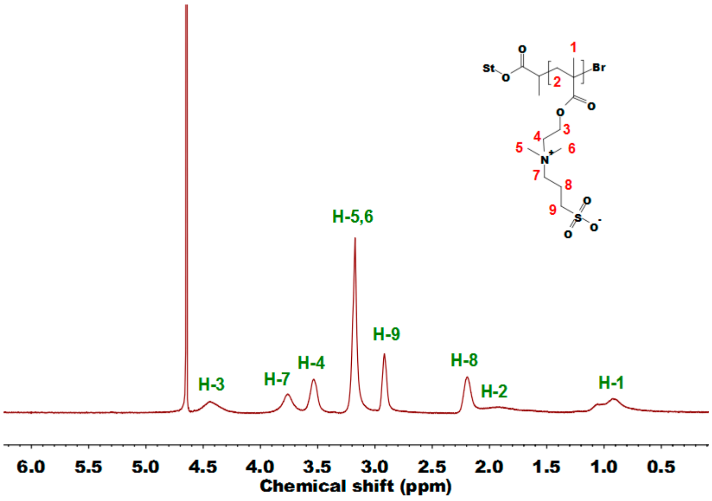

Figure S10. ^{1}H-NMR spectrum of Starch-g-PSBMA

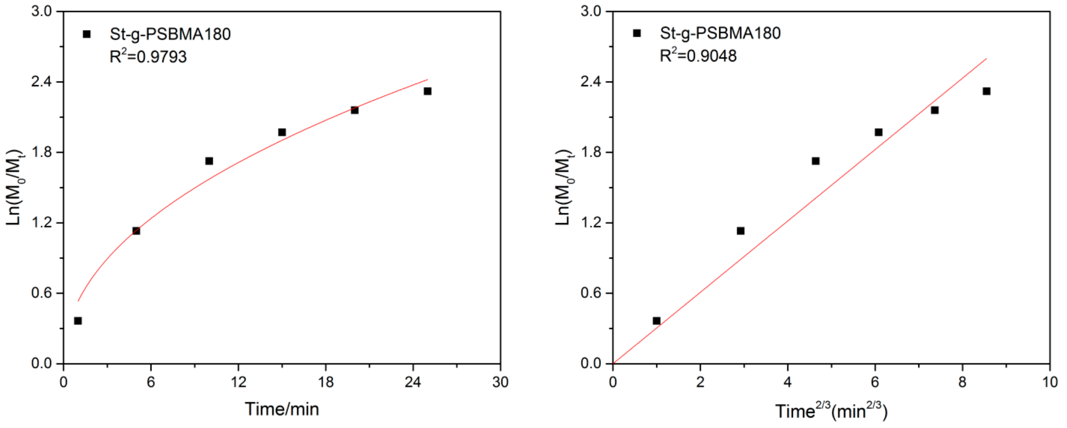

Figure S11. Kinetic plot for the synthesis of St-g-PSBMA with target DP of 200

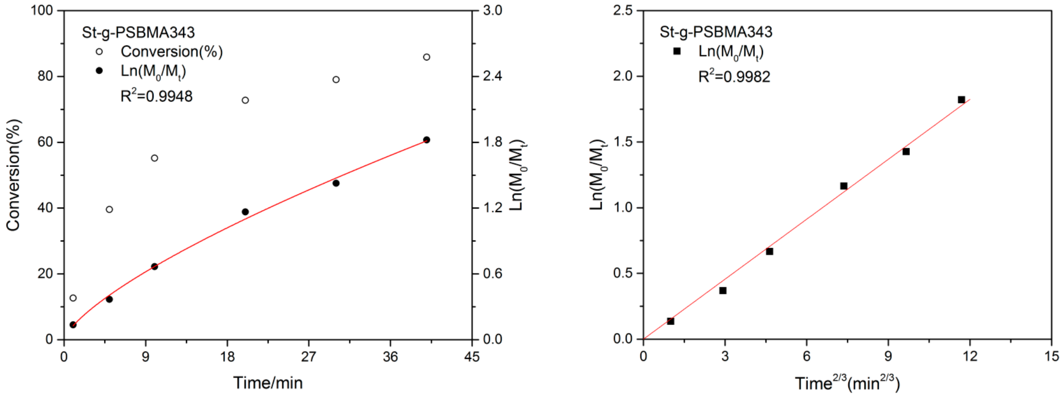

Figure S12. Kinetic plot for the synthesis of St-g-PSBMA with target DP of 400

© 2018 by the authors. Submitted for possible open access publication under the terms and conditions of the Creative Commons Attribution (CC BY) license (http://creativecommons.org/licenses/by/4.0/).
